# Incidence of Carotid Wall Irregularities in Acute Ischemic Stroke Patients: A Descriptive Analysis

**DOI:** 10.7759/cureus.70606

**Published:** 2024-10-01

**Authors:** Aparnna Unnikrishnan Nair, Nitin Ramanathan, Pritika Gnanasekaran, Rama Krishna Srirangam, Shanmukha Vinay, Kishore Sekar, Sairam Subburam, Monica Gaurdian, Yagyavalkya Sharma, Shoraf Pascal, Pratibha Wankhede, Prashanth A

**Affiliations:** 1 Internal Medicine, Altnagelvin Area Hospital, Western Health and Social Care Trust, Londonderry, GBR; 2 Biochemistry, Jagadguru Sri Shivarathreeshwara (JSS) Academy of Higher Education and Research, Mysore, IND; 3 Internal Medicine, Global Medical Center, Salem, IND; 4 Internal Medicine, Queen Elizabeth Hospital Birmingham, Birmingham, GBR; 5 Cardiology, CARE Hospitals, Visakhapatnam, IND; 6 General Medicine, SR Clinic, Perambalur, IND; 7 Cardiology, KG Heart Care Clinic, Salem, IND; 8 Emergency Medicine, University Hospitals Birmingham NHS Foundation Trust, Birmingham, GBR; 9 Life Sciences, Team MP Research Work, Mathura, IND; 10 Biotechnology, Kalp Laboratories, Mathura, IND; 11 Community Medicine, Madha Medical College and Research Institute, Chennai, IND; 12 Community Health Nursing, Shrimati Radhikabai Meghe Memorial College of Nursing, Datta Meghe Institute of Higher Education & Research, Wardha, IND; 13 Physiology, Mahatma Gandhi Institute of Medical Sciences, Wardha, IND

**Keywords:** cardiovascular risk factors, carotid wall abnormalities, hypertension, intima-media thickness, ischemic stroke, lipid profiles, smoking

## Abstract

Background

Carotid wall abnormalities are significant predictors of cardiovascular events, including ischemic stroke. Identifying the clinical and biochemical risk factors associated with these abnormalities can aid in early intervention and prevention strategies. This study aimed to assess the association between lipid profiles, age, gender, smoking habits, hypertension, and carotid wall abnormalities in patients.

Methodology

A cross-sectional study was conducted on 60 patients, evaluating their clinical and biochemical profiles, including lipid levels, age, gender, smoking status, and the presence of hypertension. Carotid intima-media thickness was measured using ultrasound to identify carotid wall abnormalities. The data were analyzed to determine the associations between these factors and the presence of carotid wall abnormalities.

Results

Carotid wall abnormalities were present in 78.3% (n = 47) of the patients. Individuals with carotid wall irregularities exhibited markedly elevated total cholesterol concentrations (175 ± 35.0 mg/dl) compared to those without abnormalities (150 ± 31.0 mg/dl) (p = 0.007). The mean age was 64.0 ± 8.0 years in the abnormality group versus 56.0 ± 5.0 years in the non-abnormality group (p = 0.008). Males constituted 80.0% of the abnormality group, compared to 46.7% in the non-abnormality group (p = 0.03). This higher prevalence of carotid wall abnormalities in males could be related to gender-specific risk factors, such as higher rates of smoking and hypertension, both of which were more common in the abnormality group and are known contributors to vascular changes. Smoking (70% vs. 20%, p = 0.0005) and hypertension (85% vs. 40%, p = 0.0005) were significantly more prevalent in individuals with carotid wall abnormalities.

Conclusions

This study highlights the significant association between elevated total cholesterol, older age, male gender, smoking, and hypertension with carotid wall abnormalities. These findings emphasize the importance of early detection and management of these risk factors to prevent the progression of carotid atherosclerosis and reduce the risk of ischemic stroke and other cardiovascular events.

## Introduction

Stroke, a cerebrovascular accident, is a leading cause of morbidity and mortality in developing nations. It can be categorized into ischemic, hemorrhagic, or subarachnoid types, with ischemic stroke being the most prevalent. The TOAST classification system further categorizes ischemic stroke into major-vessel atherosclerosis, microvascular obstruction, and stroke of indeterminate origin [1]. Ischemic strokes are often triggered by thrombotic or embolic events that reduce cerebral blood flow. Thrombosis occurs due to vessel dysfunction, commonly resulting from atherosclerosis or fibromuscular dysplasia, while embolic strokes arise from debris blocking the cerebral vessels [2,3]. The etiology of the stroke plays a crucial role in determining patient prognosis and outcomes.

Globally, stroke was the second leading cause of death in 2019, accounting for 11% of all deaths, according to the World Health Organization [4]. Annually, 15 million people suffer from stroke worldwide, with five million fatalities and another five million experiencing permanent disability. Carotid wall abnormalities play a significant role in thrombotic stroke, as they contribute to atherosclerosis and plaque formation, leading to carotid artery stenosis [5,6]. This narrowing of the carotid arteries is a critical factor in ischemic (thrombotic) strokes, which occur at a rate ten times higher than hemorrhagic strokes [7]. Studies suggest that carotid artery stenosis is responsible for approximately 10-20% of all ischemic strokes, emphasizing the importance of carotid wall health in stroke prevention. Additionally, women, who generally experience strokes at an older age, are more likely to endure recurrent stroke events [8].

Diagnostic imaging, particularly CT and MRI, plays a critical role in stroke management [9]. Despite MRI’s higher cost, CT scans are more frequently used due to their accessibility, aiding in identifying the location, extent, and characteristics of the brain lesion [10]. Carotid Doppler ultrasonography (USG) is crucial for assessing carotid intima-media thickness (CIMT), plaque characteristics, and stenosis, providing a comprehensive view of the carotid arteries' condition [11]. This technique, through B-mode ultrasound imaging, reveals atherosclerotic changes and age-related vascular alterations [12]. The intima and media layers, typical areas for lipid accumulation and plaque development, are key to understanding stroke risk [13].

In adults, a normal CIMT ranges from 0.5 to 0.9 mm, with values exceeding 0.9 mm considered abnormal, often associated with sonographically visible plaque. Although stroke prevalence has been extensively studied, information from Southern India regarding carotid arterial irregularities in ischemic stroke patients remains limited. The existing information is insufficient to guide preventive strategies and accurately assess stroke prognosis. This research was carried out to fill this void by evaluating the frequency of carotid arterial wall irregularities in individuals experiencing acute ischemic stroke and determining the related risk factors. The function of carotid Doppler imaging in identifying strokes was also examined to enhance the understanding of stroke management in this region.

## Materials and methods

Study design and setting

This observational study was conducted on a cohort of 60 patients admitted with acute ischemic stroke at Madha Medical College and Research Institute, Chennai, India. The research spanned six months, from January 2024 to July 2024. Ethical approval was obtained from the institutional ethics committee prior to the commencement of the study (MMCRI/IEC/2023/033). Informed consent was secured from each participant, ensuring adherence to ethical standards throughout the research process.

Sample Size Calculation

Based on the findings from Compagne et al. [14], which reported a prevalence of 2.5% for carotid abnormalities, including webs, in patients with acute ischemic stroke, the sample size calculation was performed using the formula N = Z²∙P∙Q/E². Here, “P” represents the prevalence (2.5%), and “Q” is 1-P. With a confidence level of 95% and a margin of error of 5%, the calculated minimum sample size was determined to be 38 patients. However, to account for potential losses to follow-up, we included 60 patients in our study to ensure the robustness of our findings.

Selection Criteria

The selection criteria for this study included patients who were above 18 years of age, regardless of gender, and had been diagnosed with an ischemic stroke confirmed by CT showing an infarct. Only those patients who consented and were capable of providing informed consent for participation were enrolled in the study. However, certain groups were excluded from the study: those with incomplete data, expectant and breastfeeding women, as well as individuals diagnosed with hemorrhagic stroke.

Data sources and variables

Data collection involved a comprehensive approach starting with demographic information, followed by obtaining the patient’s medical history to identify any comorbidities and past addictions, such as smoking. A thorough physical examination was conducted, and several key investigations were performed, including ECG assessments, CT scans to determine the area involved, and carotid Doppler examinations. During the carotid Doppler examination, patients were positioned supine, and transducer positions were employed to assess the carotid arteries in long-axis planes, capturing images of the frequent carotid arteries and the internal carotid vessels. The peak and average blood flow speeds, CIMT, and the presence of atheromatous plaques or thrombi were recorded, with the percentage of stenosis calculated. The carotid Doppler was conducted using an Esaote USG machine, ensuring precise imaging and data collection.

Statistical analysis

The data was presented as frequency distributions for categorical variables and mean ± SD for continuous variables. To assess associations between continuous variables, the independent t-test was employed. For categorical variables, the chi-square test was used. A p-value of less than 0.05 was considered statistically significant in determining the differences between groups.

## Results

Table 1 shows the age and gender distribution among the study participants. The majority of patients were in the 61-70 age group, representing 18 patients (30%), followed by the 51-60 age group with 14 patients (23.33%). The age group 41-50 included 12 patients (20%), while 11 patients (18.33%) were in the 71-80 age group. The younger age groups of 31-40 and 18-30 had lower representations, with three patients (5%) and two patients (3.33%), respectively.

**Table 1:** Age and gender distribution of patients

Age group	Frequency	Percentage (%)
18-30	2	3.33%
31-40	3	5%
41-50	12	20%
51-60	14	23.33%
61-70	18	30%
71-80	11	18.33%

Table 2 highlights the gender distribution among the patients. Out of the total, 43 patients (71.67%) were male, and 17 patients (28.33%) were female.

**Table 2:** Gender distribution

Gender	Frequency	Percentage (%)
Male	43	71.67%
Female	17	28.33%

Table 3 presents the smoking status of the patients. A total of 25 patients (41.70%) were smokers, while 35 patients (58.30%) were non-smokers.

**Table 3:** Smoking status

Smoking status	Frequency	Percentage
Smokers	25	41.70%
Non-smokers	35	58.30%

Table 4 provides an overview of the medical comorbidities observed in the patients. Hypertension (HTN) was the most common comorbidity, found in 36 patients (60%). Diabetes was present in 15 patients (25%), followed by coronary artery disease in four patients (6.67%), and hypothyroidism in three patients (5%). Additionally, 10 patients (16.67%) had both HTN and diabetes, while seven patients (11.67%) had a combination of HTN, diabetes, and CAD.

**Table 4:** Comorbidity CAD, coronary artery disease; HTN, hypertension

Comorbidity	Frequency	Percentage (%)
HTN only	36	60%
Diabetes only	15	25%
CAD only	4	6.67%
Hypothyroidism only	3	5%
HTN and diabetes combined	10	16.67%
HTN, diabetes, and CAD combined	7	11.67%

Table 5 displays the lipid profile parameters of the patients. The mean total cholesterol level was 165 ± 33.0 mg/dl, with a range of 90-170 mg/dl. Triglyceride levels had a mean of 150 ± 25.0 mg/dl, ranging from 60 to 400 mg/dl. The mean low-density lipoprotein (LDL) level was 120 ± 22.0 mg/dl, with a range of 40-200 mg/dl. High-density lipoprotein (HDL) levels averaged 32.5 ± 4.5 mg/dl, within a range of 20-50 mg/dl. Very LDL levels averaged 24.0 ± 10.0 mg/dl, with a range of 20-120 mg/dl.

**Table 5:** Lipid profile of patients HDL, high-density lipoprotein; LDL, Low-density lipoprotein; VLDL, very low-density lipoprotein

Parameter	Range	Mean ± SD
Total cholesterol (mg/dl)	90-170	165 ± 33.0
Triglycerides (mg/dl)	60-400	150 ± 25.0
LDL (mg/dl)	40-200	120 ± 22.0
HDL (mg/dl)	20-50	32.5 ± 4.5
VLDL (mg/dl)	20-120	24.0 ± 10.0

Table 6 summarizes the ECG findings among the patients. Normal sinus rhythm (NSR) was the most common finding, observed in 36 patients (60%). Sinus bradycardia was present in four patients (6.67%), while sinus tachycardia was noted in three patients (5%). ST-T wave changes were observed in 17 patients (28.33%).

**Table 6:** ECG finding NSR, normal sinus rhythm

ECG finding	Frequency	Percentage (%)
NSR	36	60%
Sinus bradycardia	4	6.67%
Sinus tachycardia	3	5%
ST-T wave changes	17	28.33%

Table 7 illustrates the areas of involvement identified through CT scans. The capsuloganglionic region was the most frequently involved area, seen in 24 patients (40%). The thalamus was involved in 15 patients (25%), while the frontal lobes were affected in 12 patients (20%). Other regions accounted for nine patients (15%).

**Table 7:** Area involved

Area involved	Frequency	Percentage (%)
Capsuloganglionic region	24	40%
Thalamus	15	25%
Frontal lobes	12	20%
Other regions	9	15%

Table 8 reveals the presence of carotid wall abnormalities among the patients. CIMT ≥0.9 mm, indicating carotid wall abnormality, was present in 47 patients (78.33%). In contrast, 13 patients (21.67%) had a CIMT <0.9 mm, indicating the absence of carotid wall abnormality.

**Table 8:** Carotid wall abnormality CIMT, carotid intima-media thickness

Carotid wall abnormality	Frequency	Percentage (%)
Present (CIMT ≥0.9 mm)	47	78.33%
Absent (CIMT <0.9 mm)	13	21.67%

Table 9 explores the associations between various parameters and carotid wall abnormalities. Patients with carotid wall abnormalities (n = 45) had a mean HDL level of 35.0 ± 3.2 mg/dl, while those without abnormalities (n = 15) had a mean HDL level of 33.0 ± 4.5 mg/dl (p = 0.14, t = 1.51). The mean triglyceride level was 135 ± 25 mg/dl in patients with carotid wall abnormalities, compared to 130 ± 21.5 mg/dl in those without (p = 0.42, t = 0.80). The mean LDL level was 110 ± 23.5 mg/dl in patients with carotid wall abnormalities, compared to 100 ± 30.0 mg/dl in those without (p = 0.21, t = 1.23). The mean total cholesterol level was significantly higher in patients with carotid wall abnormalities (175 ± 35.0 mg/dl) than in individuals without these abnormalities (150 ± 31.0 mg/dl) with a p-value of 0.007 (t = 2.84). The mean age of patients with carotid wall abnormalities was 64.0 ± 8.0 years, significantly older than the mean age of 56.0 ± 5.0 years in those without (p = 0.008, t = 2.74). Gender distribution showed that 80% of patients with carotid wall abnormalities were male, compared to 46.70% in those without (p = 0.03, χ² = 4.60). Additionally, 70% of patients with carotid wall abnormalities were smokers, compared to 20% of those without (p = 0.0005, χ² = 19.20). The presence of hypertension was significantly higher in patients with carotid wall abnormalities (85%) than in individuals without these abnormalities (40%), with a p-value of 0.0005 (χ² = 17.40).

**Table 9:** Associations with carotid wall abnormalities HDL, high-density lipoprotein; LDL, Low-density lipoprotein

Parameter	Carotid wall abnormality present (n = 45)	Carotid wall abnormality absent (n = 15)	p-value	Test used	Test statistic
HDL (mean ± SD)	35.0 ± 3.2 mg/dl	33.0 ± 4.5 mg/dl	0.14	Independent t-test	t = 1.51
Triglycerides (mean ± SD)	135 ± 25 mg/dl	130 ± 21.5 mg/dl	0.42	Independent t-test	t = 0.80
LDL (mean ± SD)	110 ± 23.5 mg/dl	100 ± 30.0 mg/dl	0.21	Independent t-test	t = 1.23
Total cholesterol (mean ± SD)	175 ± 35.0 mg/dl	150 ± 31.0 mg/dl	0.007	Independent t-test	t = 2.84
Mean age (years)	64.0 ± 8.0 years	56.0 ± 5.0 years	0.008	Independent t-test	t = 2.74
Gender (male)	80%	46.70%	0.03	Chi-square test	χ² = 4.60
Smokers	70%	20%	0.0005	Chi-square test	χ² = 19.20
Hypertension presence	85%	40%	0.0005	Chi-square test	χ² = 17.40

## Discussion

This study offers an in-depth analysis of the medical and biochemical profiles of individuals with carotid wall abnormalities, focusing on associations with lipid levels, age, gender, smoking habits, and hypertension. The observation that individuals exhibiting carotid wall irregularities showed markedly elevated levels of total cholesterol (175 ± 35.0 mg/dl) compared to those without these abnormalities (150 ± 31.0 mg/dl), with a p-value of 0.007, underscores the critical role of lipid management in this population. This finding is consistent with the research by Polak et al. [15], which demonstrated a clear link between increased CIMT and elevated cholesterol levels, thereby increasing the risk of cardiovascular diseases. The implication of this is significant; addressing elevated cholesterol levels in patients with carotid wall abnormalities could potentially mitigate their cardiovascular risk.

Interestingly, while the total cholesterol levels showed a significant difference, other lipid parameters, including HDL, triglycerides, and LDL, did not demonstrate statistically significant variations between the two groups. This suggests that total cholesterol may be a more reliable indicator for identifying patients at risk for carotid wall abnormalities, aligning with clinical practices that prioritize total cholesterol screening in vascular assessments. The mean age of patients with carotid wall abnormalities was 64.0 ± 8.0 years, significantly higher than the 56.0 ± 5.0 years observed in those without abnormalities (p = 0.008). This finding aligns with the 15 cities young stroke study by Putaala et al. [16], which identified older age as a significant risk factor for carotid wall abnormalities and subsequent ischemic events. The higher prevalence of carotid wall abnormalities in individuals in their 60s may be attributed to cumulative exposure to risk factors such as smoking, hypertension, and hyperlipidemia, as well as aging-related vascular changes that contribute to plaque formation.

Moreover, gender differences were evident, with 80% of patients with carotid wall abnormalities being male compared to 46.7% without abnormalities (p = 0.03). This gender disparity highlights the need for targeted interventions in at-risk male populations, as suggested by prior research indicating that males are more likely to develop cardiovascular conditions due to lifestyle and biological factors. The study also highlights the importance of cardiac function, as evidenced by the ECG findings. NSR was the most prevalent ECG finding, observed in 36 patients (60%). However, significant deviations, such as ST-T wave changes noted in 17 patients (28.33%), warrant attention as they may indicate underlying cardiac issues such as ischemia. This correlation is crucial; the coexistence of carotid artery disease and cardiac abnormalities can significantly impact overall cardiovascular risk. The presence of ST-T wave changes may suggest the need for further cardiac evaluation and intervention in patients with carotid wall abnormalities. Furthermore, a significant association was found between smoking and carotid wall abnormalities, with 70.0% of smokers exhibiting these abnormalities compared to 20.0% of non-smokers (p = 0.0005). Similarly, hypertension was more prevalent in patients with carotid wall abnormalities (85.0%) compared to those without (40.0%), also with a p-value of 0.0005. These findings echo the results from Sahoo et al. [17], who highlighted smoking and hypertension as critical risk factors for the development of carotid arterial irregularities, especially among individuals experiencing ischemic stroke.

The high prevalence of carotid wall abnormalities among the study population, particularly in relation to modifiable risk factors like smoking and hypertension, underscores the importance of early detection and management. This conclusion is in line with the recommendations from Yi et al. [18], who emphasized the need for population-based strategies to reduce the burden of stroke and associated risk factors. Addressing comorbidities is vital, as evidenced by Johansen et al. [19], who reported on the significant impact of hypertension and diabetes on stroke outcomes. The distribution of stroke risk factors based on stroke subtypes observed in this study further parallels the findings from Habibi-Koolaee et al. [20], underscoring the need for personalized approaches to patient care. Finally, the observed correlation between increased CIMT and cardiovascular disease risk, as reported by Sarika and Kumar [21], further supports the necessity for integrated management approaches targeting both CIMT and modifiable risk factors in this patient population. The findings of this study emphasize the critical role of comprehensive cardiovascular assessments and the implementation of lifestyle modifications to improve patient outcomes.

Limitations of the study

This research has various constraints that need to be recognized. Firstly, the snapshot approach restricts the capacity to determine causal connections between carotid wall abnormalities and the related risk factors. Additionally, the research was carried out at one location, which might limit the applicability of the results to wider populations. The number of participants, though adequate for detecting significant associations, may still limit the ability to explore less common risk factors. Another limitation is the reliance on patient self-reporting for certain variables, such as smoking habits and medical history, which could introduce recall bias. Furthermore, the study did not account for potential confounding factors, such as physical activity levels or dietary habits, which might influence the observed relationships. Finally, the use of ultrasound for measuring CIMT provides valuable insights but may lack the precision of more advanced imaging techniques. Despite these limitations, the study offers important contributions to understanding the factors associated with carotid wall abnormalities.

## Conclusions

This study provides valuable insights into the clinical and biochemical characteristics associated with carotid wall abnormalities in patients. The findings underscore the significant role that elevated total cholesterol levels, advanced age, male gender, smoking, and hypertension play in the development of carotid wall abnormalities. These risk factors, which are largely modifiable, emphasize the urgent necessity for focused preventative strategies and prompt actions to mitigate the progression of carotid atherosclerosis. The observed associations between carotid wall abnormalities and these factors are consistent with existing literature, reinforcing the importance of comprehensive cardiovascular risk management. By addressing these key risk factors, it may be possible to reduce the incidence of ischemic stroke and other cardiovascular events in at-risk populations. Notwithstanding its constraints, this research contributes to the growing repository of proof endorsing the amalgamation of routine CIMT assessments into clinical practice, particularly for patients with multiple cardiovascular risk factors. Such proactive approaches could play a pivotal role in enhancing enduring results and elevating the living standards for patients at risk of cerebrovascular diseases.
